# Epigenomics-Guided Multi-Omics Integration Uncovers a Lipid-Metabolic Signature with Translational Utility in Bladder Cancer

**DOI:** 10.34133/csbj.0139

**Published:** 2026-07-24

**Authors:** Yu-De Wang, Yo-Liang Lai, Chia-Hsin Liu, Yu-Ting Su, Pei-Chun Shen, Fang-Hsin Chen, Chia-Yang Li, Shu-Chi Wang, Wen-Lung Ma, Chi-Ping Huang, Wei-Chung Cheng

**Affiliations:** ^1^ Department of Urology, China Medical University Hospital, Taichung, Taiwan.; ^2^ Graduate Institute of Biomedical Sciences, China Medical University, Taichung, Taiwan.; ^3^ Department of Radiation Oncology, China Medical University Hospital, Taichung, Taiwan.; ^4^ School of Medicine, China Medical University, Taichung, Taiwan.; ^5^ Cancer Biology and Precision Therapeutics Center, China Medical University, Taichung, Taiwan.; ^6^ Department of Obstetrics and Gynecology, Department of Medical Research, China Medical University Hospital, Taichung, Taiwan.; ^7^ Institute of Nuclear Engineering and Science, National Tsing Hua University, Hsinchu, Taiwan.; ^8^ Graduate Institute of Medicine, College of Medicine, Kaohsiung Medical University, Kaohsiung, Taiwan.; ^9^ Department of Medical Laboratory Science and Biotechnology, Kaohsiung Medical University, Kaohsiung, Taiwan.; ^10^ Department of Medical Research, Organ Transplantation Center, China Medical University Hospital, Taichung, Taiwan.; ^11^ Ph.D. Program for Health Science and Industry, Center of Tumor Biology, School of Medicine, China Medical University, Taichung, Taiwan.; ^12^ Office of Research and Development, Asia University, Taichung, Taiwan.; ^13^ Program for Cancer Biology and Drug Discovery, China Medical University, Taichung, Taiwan.

## Abstract

**Background:** Bladder cancer (BLCA) exhibits marked heterogeneity, and current classifiers provide limited guidance for prognosis or treatment. Because epigenetic reprogramming and metabolic rewiring jointly shape BLCA biology, we sought to identify epigenomically informed biomarkers with functional relevance. **Methods:** Epigenome (genome-wide promoter DNA methylation) and matched transcriptome (RNA sequencing) profiles from tumor and adjacent normal samples were integrated to identify genes with concordant differential methylation and expression patterns. A survival-oriented machine learning framework distilled these candidates into a 25-gene signature. The prognostic performance was evaluated in 4 independent BLCA cohorts. Multilayer characterization included the computational inference of tumor-infiltrating immune cells and *in silico* drug sensitivity prediction. The functional relevance of key lipid metabolic hub genes was confirmed by pharmacological inhibition in BLCA cell line models, followed by colony formation and migration assays. **Results:** The signature, enriched for cell cycle regulation and lipid metabolism, stratified patients into high- and low-risk groups across the discovery and 4 validation datasets. The prognostic value remained independent of age, pathological stage, and common genomic alterations. Low-risk tumors exhibited computationally inferred immune-inflamed phenotypes, whereas high-risk tumors exhibited lower immune engagement and lower half-maximal inhibitory concentration values for several drugs. Network analysis identified fatty acid synthase and stearoyl-coenzyme A desaturase as central nodes; their inhibition reduced BLCA cell proliferation and migration, supporting pathway-level functional relevance. **Conclusion:** By integrating epigenomic and transcriptomic layers with machine learning, we delineated a lipid-centric 25-gene signature that delivers stage-independent prognostication, illuminates tumor–immune interactions, and nominates actionable therapeutic targets. This experimentally vetted multi-omics framework advances precision oncology for BLCA.

## Graphical Abstract

**Figure:**
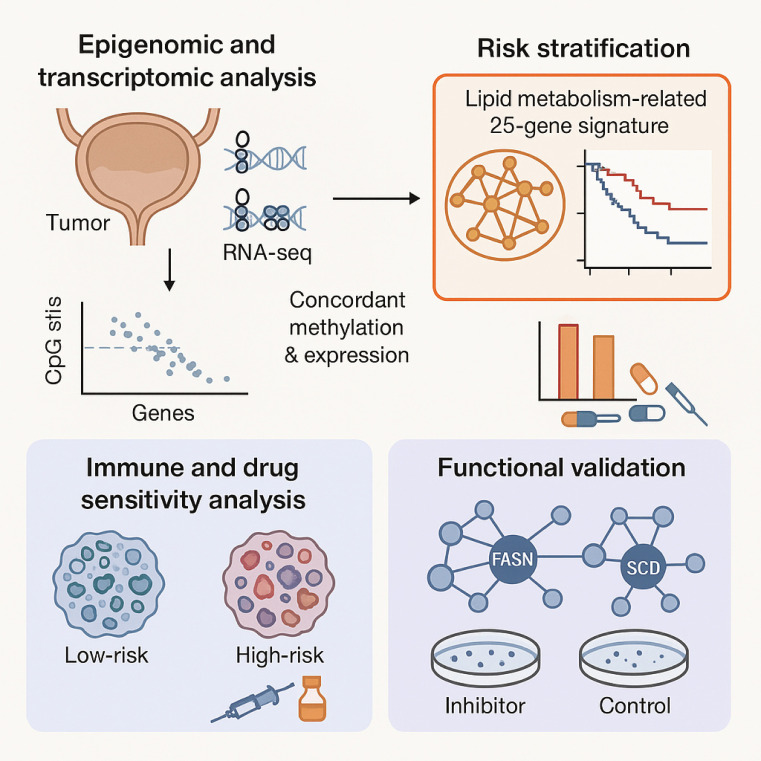

## Introduction

Bladder cancer (BLCA) is a major global health challenge, ranking among the 10 most prevalent cancers, with approximately 573,000 new cases and 213,000 deaths annually [1]. Approximately 75% of cases present as nonmuscle-invasive bladder cancer (NMIBC) and are managed using transurethral resection of the bladder tumor followed by intravesical therapy, whereas muscle-invasive bladder cancer (MIBC) requires radical cystectomy (RC) and systemic treatment [2,3]. Despite these strategies, the prognosis remains poor, with NMIBC recurring in 57% to 67% of cases and progressing in 7% to 46% of cases. Untreated MIBC has a 5-year survival rate of less than 15%, and even with aggressive therapy, approximately 50% of patients develop metastases, with a median survival of 14 to 31.5 months [3–6]. The molecular heterogeneity of BLCA further complicates decisions regarding intravesical schedules, early RC in high-risk NMIBC, and adjuvant therapy for MIBC. Although several molecular classifiers, such as those developed by The Cancer Genome Atlas (TCGA), University of North Carolina, and MD Anderson Cancer Center, have been proposed, they mainly reflect genetic clustering without robust associations with clinical outcomes, limiting their utility in guiding treatment decisions [7,8]. Even conventional clinical tools, such as tumor node metastasis staging and the World Health Organization grading system, although widely used to guide treatment, offer only approximately 70% accuracy, with frequent under- and overstaging, and demonstrate considerable variability in prognostic value [9,10]. Key driver genes, such as fibroblast growth factor receptor 3 (*FGFR3*) and tumor protein p53 (*TP53*), have also been linked to tumorigenesis and prognosis [8,11]. However, targeted therapy development remains limited, with *FGFR3* inhibitors being the only Food and Drug Administration (FDA)-approved biomarker-driven treatment, benefiting only 10% to 20% of patients with MIBC [12]. These limitations underscore the urgent need for novel and reliable biomarkers to support personalized treatment strategies, minimize the risk of over- or undertreatment, and facilitate the discovery of new therapeutic options for this patient population.

DNA methylation is a critical epigenetic modification intricately involved in BLCA biology, substantially influencing tumor initiation, progression, and prognosis [13]. Predominantly occurring at CpG islands within gene promoter regions, aberrant DNA methylation frequently results in transcriptional silencing of tumor suppressor genes, such as cadherin 13 (*CDH13*), RB binding protein 8, endonuclease (*RBBP8*), and inter-alpha-trypsin inhibitor heavy chain family member 5 (*ITIH5*), contributing substantially to BLCA tumorigenesis [14–16]. *CDH13* hypermethylation is strongly associated with aggressive tumor phenotypes and poor survival outcomes [16]. Moreover, DNA methylation affects immune regulation and treatment responses [17]. Methylation mediated by methyltransferase-like 3 (*METTL3*) promotes BLCA’s escape from the immune system [18]. Clinically, methylation, urinary microRNA, and plasma circulating tumor DNA tests outperform cytology and cystoscopy in BLCA detection and surveillance [19]. In addition to methylation, energy metabolism reprogramming is a hallmark of cancer. Notably, lipid metabolism is altered in highly proliferative cells, and the activation of fatty acid synthesis emerges as a key facet of oncogenic signaling pathways in cancer [20]. In BLCA, accumulating evidence indicates that shifts in lipid metabolism considerably influence the energy balance and immune-related pathways [21,22]. Altered lipid metabolism and methylation-based epigenetic regulation drive BLCA tumor growth and immune modulation, highlighting promising diagnostic, prognostic, and therapeutic avenues that merit clinical investigation [19].

This study aimed to address the critical gap in BLCA management arising from the lack of robust theranostic markers. Specifically, the primary objective was to identify and validate potential biomarkers and therapeutic targets, emphasizing the prognostic significance of a lipid-related gene signature. By integrating this gene signature with established clinical variables, we sought to develop a comprehensive and clinically applicable prognostic tool to guide therapeutic decision-making. Furthermore, we explored potential therapeutic strategies via *in silico* drug discovery based on this gene signature. Another objective was to experimentally elucidate how changes in lipid metabolism affect fundamental oncogenic processes in BLCA cell lines. Ultimately, these combined approaches promise to enhance personalized treatment strategies and advance our understanding of lipid biology in BLCA.

## Results

### Integrative transcriptomic and methylomic analysis reveals a robust prognostic gene signature for BLCA

**Fig. 1.**
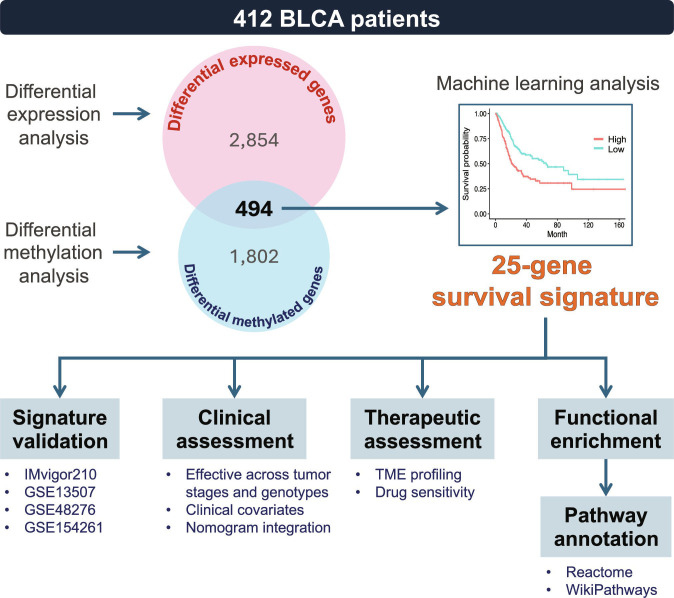
Workflow of the integrated multi-omics approach to identifying a prognostic gene signature in bladder cancer (BLCA). Initial analyses of differential expression and methylation using The Cancer Genome Atlas (TCGA)-BLCA data identified 494 overlapping genes. These were further refined using a machine learning random forest model, resulting in a robust 25-gene survival signature that stratified patients into high- and low-risk groups. Subsequent validation steps included clinical assessment, therapeutic prediction, and functional enrichment analyses.

**Fig. 2.**
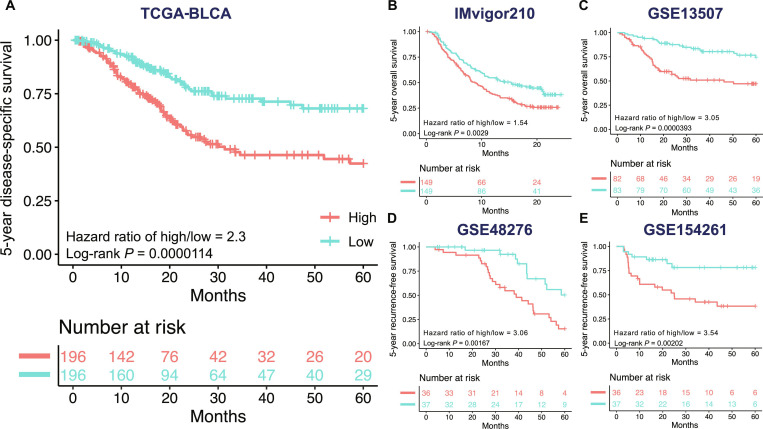
Prognostic performance of the 25-gene signature across discovery and validation cohorts. Kaplan–Meier survival analyses of the 25-gene risk score in the training cohort (The Cancer Genome Atlas [TCGA]-bladder cancer [BLCA] (A) and four independent BLCA cohorts (B to E).

An integrative analysis of the TCGA dataset identified 494 genes with concordant differential expression and methylation (Fig. 1 and Fig. S1). From these, a survival-oriented random forest model distilled a robust 25-gene prognostic signature (Table S1). To confirm the functional impact of epigenetic regulation, we verified that these genes exhibited significant inverse correlations between promoter methylation and gene expression (Spearman’s correlation test, *P* < 0.05), consistent with the selection criteria of the MethylMix algorithm (Fig. S2). This signature demonstrated significant power in predicting the disease-specific survival of patients with BLCA (hazard ratio [HR] = 2.3, *P* = 1.14 × 10^−5^) (Fig. 2A). The details of the 25 genes in this model are presented in Table S1.

In the discovery cohort, high-risk patients exhibited significantly worse overall survival (OS) (Fig. 2B to E) than low-risk patients. This prognostic value was validated across 4 independent cohorts, confirming poor outcomes for high-risk patients in metastatic (IMvigor210, HR = 1.54), NMIBC (GSE13507, HR = 3.05), and MIBC (GSE48276, HR = 3.06) settings, as well as reduced recurrence-free survival in Bacille Calmette–Guérin (BCG)-treated patients (GSE154261, HR = 3.54). Collectively, these data confirm the robustness and broad clinical applicability of the 25-gene signature, highlighting its consistent prognostic value for diverse patient populations, different treatment strategies (RC, chemotherapy, and BCG therapy), and varying disease stages, positioning it as a potential cornerstone for the personalized management of BLCA.

### Clinical utility and prognostic impact of the 25-gene signature across stages and genetic subtypes of BLCA

**Fig. 3.**
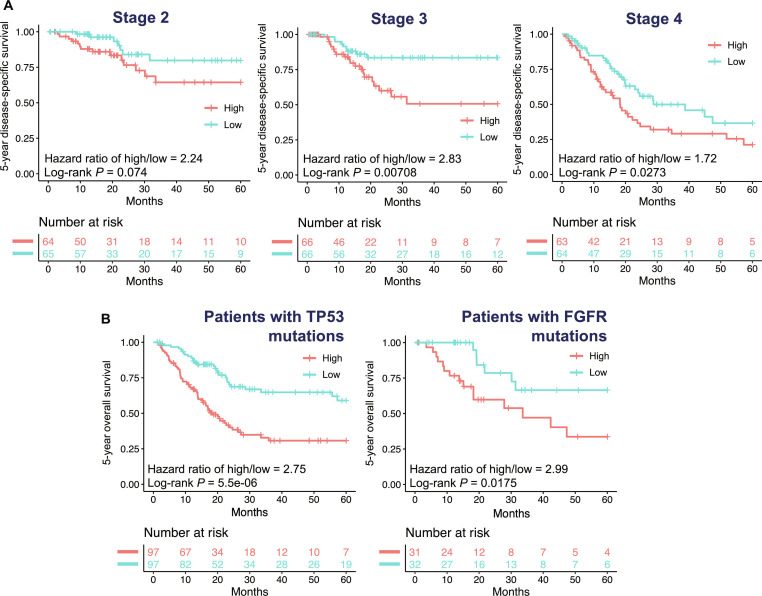
Stage- and gene-subtype-specific impact and clinical utility of the 25-gene signature. (A) Stage-stratified Kaplan–Meier curves showing the prognostic impact of the 25-gene risk score in stage 2 (hazard ratio [HR] = 2.24), stage 3 (HR = 2.83), and stage 4 (HR = 1.72) bladder cancer (BLCA). (B) Genotype-stratified survival analyses within key gene-defined subtypes, including fibroblast growth factor receptor 2/3 (*FGFR2/3*)-mutant (HR = 2.75) and tumor protein 53 (*TP53*)-mutant (HR = 2.99) tumors, demonstrating that the high-risk group has consistently worse outcomes across different genetic subtypes.

**Fig. 4.**
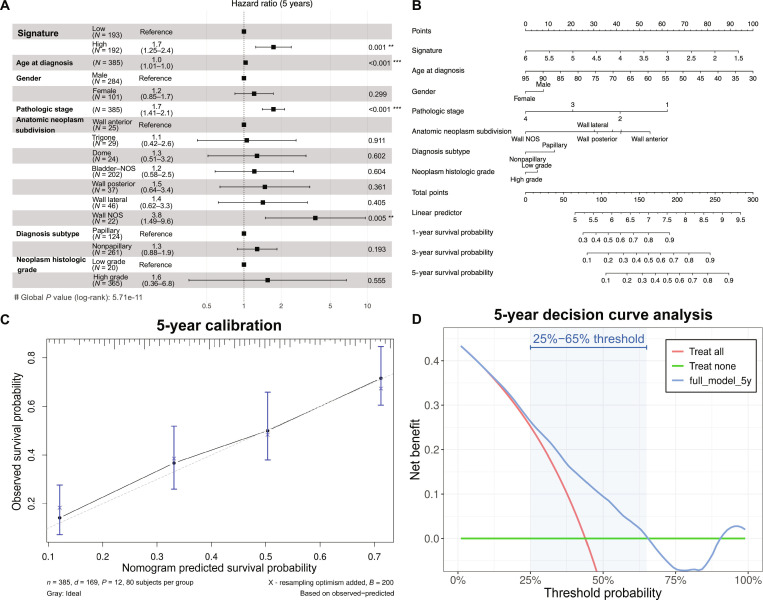
(A) Multivariable Cox regression forest plot showing that the 25-gene risk score remains an independent prognostic factor after adjustment for clinical variables. NOS, not otherwise specified. (B) Nomogram integrating the 25-gene risk score with age, sex, tumor stage, and diagnosis subtype to estimate 1-, 3-, and 5-year survival probabilities. (C) Five-year calibration plots of the nomogram. The black solid line represents the calibration curve, and the gray dashed line indicates the ideal 45° line of perfect calibration. The blue vertical bars represent 95% confidence intervals. The X symbols denote optimism-corrected estimates based on 200 bootstrap resamples. The nomogram was constructed on the basis of a Weibull parametric survival model incorporating the lipid-related signature and key clinicopathological variables. (D) The decision curve evaluates the clinical usefulness and net benefit of the integrated model (full_model_5y, blue line), which includes the risk signature and clinical covariates (age, gender, pathologic stage, anatomic subdivision, diagnosis subtype, and histologic grade) in predicting 5-year outcomes. The horizontal green line represents the strategy of treating no patients (“Treat none”, net benefit = 0), and the solid red line represents the strategy of treating all patients (“Treat all”). The *x* axis indicates the threshold probabilities, and the *y* axis represents the calculated net benefit of the model. The prognostic model offers a superior net benefit over default strategies within a threshold probability range of approximately 25% to 65%.

The 25-gene signature effectively stratified patients with BLCA across different clinical stages and genetic subtypes, demonstrating its robust prognostic value. Kaplan–Meier analyses revealed that the high-risk classification based on this gene signature was associated with worse survival outcomes. Specifically, the HR of patients identified as high-risk in stage 2 BLCA was 2.24 (*P* = 0.074), whereas the prognostic impact increased notably in stage 3 (HR = 2.83, *P* = 0.00708). Although still significant, the prognostic strength was slightly diminished in stage 4 (HR = 1.72, *P* = 0.0273) (Fig. 3A). Further evaluation of the molecular subgroups underscored the broad relevance of the signature, particularly in patients harboring *TP53* and *FGFR*2/3 mutations. The 5-year OS of high-risk *TP53*-mutant patients was markedly worse (HR = 2.75, *P* < 0.00001), whereas high-risk *FGFR*2/3-mutant patients also showed a substantial survival disadvantage (HR = 2.99, *P* = 0.0175) (Fig. 3B). Multivariate HR analyses further supported the clinical applicability of this signature, showing that the 5-year OS of high-risk patients was significantly poorer (HR = 1.7, *P* < 0.001), with age at diagnosis (HR = 1.03, *P* < 0.001) and pathological stage (HR = 1.7, *P* < 0.001) being strong predictors (Fig. 4A). Notably, gender, diagnosis subtype, and histological grade did not have significant predictive values (Fig. 4A).

To facilitate clinical decision-making, we developed a comprehensive prognostic nomogram, incorporating the 25-gene signature, patient age, gender, tumor stage, and diagnosis subtype to provide individualized predictions for 1-, 3-, and 5-year OS outcomes, thereby enhancing the precision of patient risk stratification and treatment strategies (Fig. 4B). Notably, the 5-year OS calibration plots showed good agreement between the predicted and observed probabilities, closely following the ideal line with only minor deviations in the low-risk group (Fig. 4C). Decision curve analysis was used to determine whether guiding clinical interventions using our 5-year multivariate prognostic model would improve patient outcomes compared with standard default strategies. As shown in the decision curve (blue line in Fig. 4D), the prognostic model demonstrated an evident clinical net benefit over both the “treat all” and “treat none” strategies across a wide and clinically relevant threshold probability window, specifically between 25% and 65%. Within this range, using the model to guide clinical decision-making yields a higher net benefit, indicating that the model can effectively assist in identifying high-risk patients who would benefit most from intervention while safely sparing low-risk patients from unnecessary overtreatment.

### Functional characterization of the 25-gene signature reveals associations with tumor microenvironment, drug sensitivity, and lipid metabolism in BLCA

**Fig. 5.**
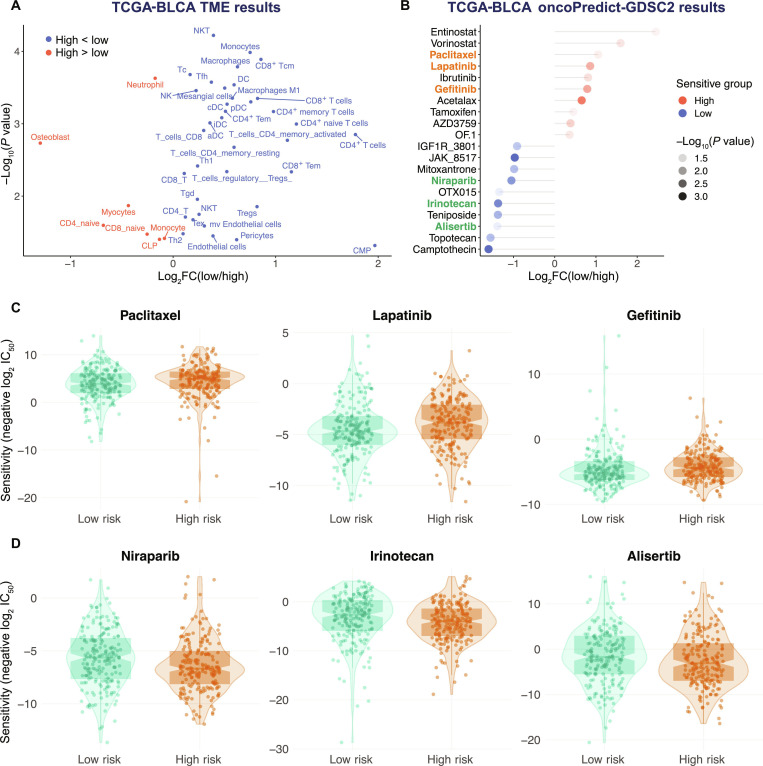
Functional characterization of the 25-gene signature. (A) Tumor microenvironment analysis comparing immune cell infiltration between the high- and low-risk groups. Tcm, central memory T cells; Tc, cytotoxic T cells; Tfh, T follicular helper cells; DC, dendritic cells; pDC, plasmacytoid dendritic cells; Tem, effector memory T cells; iDC, immature dendritic cells; cDC, conventional dendritic cells; aDC, activated dendritic cells; Th1, type 1 T helper cells; Tgd, gamma delta T cells; Tregs, regulatory T cells; Tex, exhausted T cells; CLP, common lymphoid progenitor; CMP, common myeloid progenitor. (B) Drug sensitivity prediction highlighting significantly different sensitivities to anticancer compounds between the risk groups. (C and D) Box plots illustrating differences in predicted drug sensitivity (negative log_2_ half-maximal inhibitory concentration [IC_50_] values) to selected agents, with high-risk patients sensitive to paclitaxel, lapatinib, and gefitinib, and low-risk patients sensitive to irinotecan, alisertib, and niraparib.

To comprehensively evaluate the biological relevance and therapeutic potential of the 25-gene signature, we analyzed its association with the tumor microenvironment (TME) composition and *in silico* drug response profiles of BLCA. As an exploratory analysis of TME differences, we compared the inferred immune cell populations between the risk groups (Fig. 5A). Across methods, low-risk tumors were consistently enriched for multiple CD4^+^ and CD8^+^ T cell subsets, including central memory and effector T cells as well as natural killer (NK) and natural killer T (NKT) cells, whereas high-risk tumors showed higher levels of macrophage- and fibroblast-related populations, including cancer-associated fibroblasts. The results were reported as exploratory trends rather than confirmatory findings. Interpreted cautiously, these exploratory patterns suggest a shift from a T-cell-enriched microenvironment in the low-risk group toward a myeloid- and stroma-enriched microenvironment in the high-risk group, which we regard as hypothesis-generating signals consistent with—but not confirmatory of—a possible contribution of differential immune surveillance to the better outcome of low-risk patients.

oncoPredict-based *in silico* drug response analyses revealed differentially predicted vulnerabilities based on gene signature risk classification (Fig. 5B). High-risk patients with BLCA showed lower inferred half-maximal inhibitory concentration (IC_50_) values for paclitaxel, lapatinib, ibrutinib, tamoxifen, and vorinostat, suggesting predicted drug vulnerabilities in this subgroup (Fig. 5C). In contrast, low-risk patients demonstrated lower inferred IC_50_ values for irinotecan, alisertib, and niraparib, indicating that these agents are computationally predicted to show preferential activity in tumors with a more immune-active phenotype (Fig. 5D). These findings offer preliminary biology-guided hypotheses for personalization rather than actionable treatment recommendations.

**Fig. 6.**
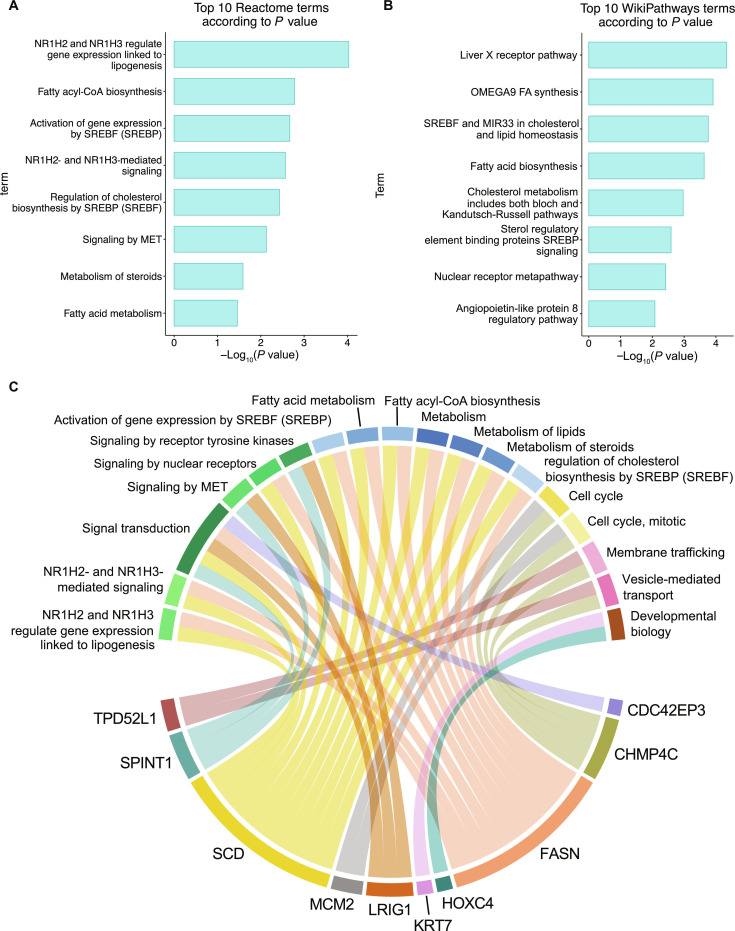
Pathway enrichment analysis of the 25-gene signature. (A) Reactome enrichment analysis identified significant pathways, including fatty acid metabolism, cholesterol biosynthesis, and nuclear receptor signaling. (B) WikiPathways analysis corroborated the involvement of these genes in lipid metabolism pathways, including *SREBF* and *MIR33* (microRNA 33) in cholesterol and lipid homeostasis. (C) Gene–pathway interaction network highlighting the central roles of lipid metabolism regulators fatty acid (FA) synthase (*FASN*) and stearoyl-coenzyme A (CoA) desaturase (*SCD*) and other genes involved in the cell cycle and membrane trafficking, including TPD52 like 1 (*TPD52L1*), leucine-rich repeats and immunoglobulin-like domains 1 (*LRIG1*), keratin 7 (*KRT7*), homeobox C4 (*HOXC4*), and CDC42 effector protein 3 (*CDC42EP3*).

Functional enrichment analyses further delineated the biological processes underlying the prognostic signature, revealing significant associations with lipid metabolism, nuclear receptor signaling, and cell cycle regulation pathways, as identified by the Reactome and WikiPathways databases. Reactome analysis highlighted key metabolic pathways, such as *NR1H2* (nuclear receptor subfamily 1 group H member 2; liver X receptor β [LXRβ])- and *NR1H3* (nuclear receptor subfamily 1 group H member 3; LXRα)-regulated gene expression, fatty acyl-coenzyme A (CoA) biosynthesis, sterol regulatory element-binding protein (SREBP)-mediated lipid regulation, MET (MET proto-oncogene, receptor tyrosine kinase) signaling, and steroid metabolism (Fig. 6A). Similarly, WikiPathways enrichment emphasized the LXR pathway, fatty acid biosynthesis, cholesterol metabolism, and SREBP signaling (Fig. 6B). Moreover, gene–pathway interaction network analysis identified fatty acid synthase (*FASN*) and stearoyl-CoA desaturase (*SCD*) as central nodes interconnected with multiple lipid-related processes, whereas other signature genes, such as *SPINT1* (serine peptidase inhibitor, Kunitz type 1), *MCM2* (minichromosome maintenance complex component 2), and *CHMP4C* (charged multivesicular body protein 4C), were implicated in signal transduction, cell cycle regulation, and membrane trafficking pathways (Fig. 6C). Collectively, these analyses underscore the critical involvement of lipid metabolism and nuclear receptor pathways in BLCA pathogenesis, positioning FASN and SCD as promising therapeutic targets for this malignancy.

### Prognostic relevance of lipid metabolism regulators *FASN* and *SCD* for BLCA and their functional validation

**Fig. 7.**
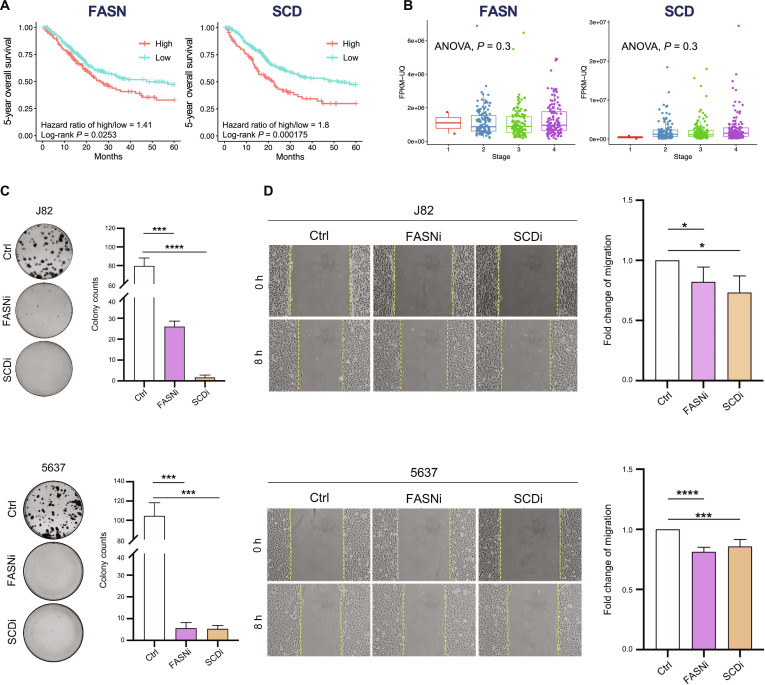
Prognostic validation and functional assessment of lipid metabolism regulators, fatty acid synthase (*FASN*) and stearoyl-coenzyme A desaturase (*SCD*). (A) Kaplan–Meier survival analysis indicating the prognostic significance of high expression levels of *FASN* (hazard ratio [HR] = 1.41) and *SCD* (HR = 1.8) in patients with bladder cancer (BLCA). (B) Expression analysis across stages revealed no significant variation (ANOVA, *P* = 0.3). (C) Colony formation assays demonstrating significantly reduced proliferation in BLCA cell lines (J82 and 5637) treated with the FASN inhibitor (FASNi; TVB-3664) and SCD inhibitor (SCDi; A939572). (D) Migration assays confirmed significantly impaired motility upon FASN and SCD inhibition, underscoring their roles in tumor aggressiveness. *P < 0.05, **P < 0.01, ***P < 0.001, and ****P < 0.0001.

To evaluate the prognostic significance of *FASN* and *SCD* in BLCA and their biological functions, we performed survival analyses, gene expression profiling, and functional assays. Kaplan–Meier survival curves for the TCGA BLCA cohort demonstrated that elevated expression of both *FASN* and *SCD* was correlated significantly with reduced OS, highlighting their prognostic potential (Fig. 7A). Specifically, high *FASN* expression was associated with an increased HR (1.41, *P* = 0.0253), whereas high *SCD* expression was an even stronger prognostic indicator (HR = 1.8, *P* = 0.0001715). Interestingly, expression analysis across tumor stages revealed no significant variation (analysis of variance [ANOVA], *P* = 0.3) (Fig. 7B), suggesting that the up-regulation of these genes occurs consistently throughout the progression of BLCA. *In vitro* functional validation using colony formation assays with the BLCA cell lines J82 and 5637 further demonstrated that treatment with FASN (TVB-3664) and SCD (A939572) inhibitors significantly inhibited cell proliferation (*P* < 0.001 for both genes; Fig. 7C). In addition, migration assays confirmed the involvement of FASN and SCD in enhancing tumor cell motility, with their inhibition leading to significantly decreased migration of both J82 (*P* < 0.05) and 5637 (*P* < 0.0001) cells (Fig. 7D). Collectively, these findings substantiate the roles of FASN and SCD as critical regulators of BLCA aggressiveness, underscoring their value as promising therapeutic targets and prognostic biomarkers.

## Discussion

This study marks a important advance in the management of BLCA by identifying and validating a robust 25-gene signature derived from integrative differential expression and differential methylation analyses. By thoroughly evaluating this signature in 4 independent cohorts encompassing various BLCA stages, our findings offer clinically relevant insights into addressing critical unmet needs in patient stratification. Incorporating this gene signature into a prognostic nomogram, alongside established clinical parameters, enhances precision in clinical decision-making, thereby mitigating the risks of over- and undertreatment. Importantly, the predictive capacity of the 25-gene signature extends beyond prognosis and serves as a valuable tool for discovering therapies and guiding personalized treatment strategies. Further biological exploration underscored the central roles of the lipid-metabolism-associated genes *FASN* and *SCD*, whose prognostic relevance and functional significance were substantiated through rigorous *in silico* and *in vitro* analyses. Collectively, these findings highlight the considerable translational potential of the 25-gene signature, positioning it as an essential resource for improved patient stratification, targeted therapeutic intervention, and a deeper mechanistic understanding of the role of lipid metabolism in BLCA progression.

Current biomarkers for BLCA primarily involve molecular classifications derived from genetic profiling, such as those reported by TCGA, distinguishing luminal and basal-squamous subtypes [23]. Despite their biological robustness, these classifications have limited clinical applicability [7], likely because molecular subtypes are established through genetic clustering rather than direct associations with clinical outcomes. In contrast, this study introduces a prognostically driven, machine-learning-derived 25-gene signature specifically optimized for clinical relevance, addressing the shortcomings of current molecular subtyping approaches. Established genetic drivers, particularly *FGFR3* and *TP53* mutations, play key roles in the pathogenesis and molecular classification of BLCA [11,19]. *FGFR3* mutations are predominantly associated with low-grade, noninvasive tumors, whereas *TP53* mutations are characteristic of high-grade, invasive tumors, underpinning the luminal and basal classifications, respectively [8]. In contrast to the BLCA immune prognostic index (BCIPI) proposed by Bien *et al.* [24], which distinguishes BCIPI-high and BCIPI-low tumors by *FGFR3* mutation patterns and corresponding microenvironmental differences, our 25-gene signature retains prognostic power irrespective of *FGFR3* or *TP53* mutation status, offering a complementary perspective on BLCA progression. Other prognostic signatures, such as the 12-gene signature of Abudurexiti *et al*. [25], are mainly statistical constructs without functional validation, which limits their biological interpretability. In contrast, the 25-gene signature in our study was validated using both *in silico* analyses and *in vitro* experiments. Importantly, the predictive strength of the 25-gene signature nomogram was consistent with recognized clinical prognostic factors. Furthermore, we compared the prognostic performance of our 25-gene signature with previously published signatures (e.g., metabolism-related gene signature by Li *et al.* [26] and lipid-metabolism-related gene signature by Zhu *et al.* [27]) and found that our 25-gene signature showed superior discrimination and achieved broader and more consistent validation across all 4 independent cohorts (Fig. S3). Specifically, older female patients with high-grade, advanced-stage, and nonpapillary tumor subtypes demonstrated higher mortality risks, consistent with existing evidence [2,3]. Consistent with contemporary evidence from artificial intelligence (AI)-enabled multi-omics approaches to BLCA prognosis [28], the integration of our gene signature into a full nomogram meaningfully increases prognostic accuracy and offers a holistic stratification scheme that merges molecular data with clinical variables. Moreover, our results support the construction of transparent, evidence-anchored tools and platforms, providing a template for structured knowledge integration that could be leveraged to externally benchmark and iteratively refine our 25-gene predictor in prospective settings [29].

The 25-gene signature provides practical value for both clinicians and researchers by enabling risk stratification applicable across disease stages, with external validation in NMIBC (Fig. 2C), T1 NMIBC on BCG (Fig. 2E), MIBC treated with chemotherapy or RC (Fig. 2D), and metastatic disease (Fig. 2B). In clinical practice, the score can be operationalized with prespecified cutoff points, a nomogram, and decision curve analysis to complement the guideline variables. Clinically, this 5-year prognostic model is not intended to infer drug-specific responsiveness but to support risk-stratified management under the current standard of care therapies. Using a prespecified threshold, such as a 60% predicted 5-year event probability, patients can be stratified into a high residual-risk group warranting consideration of treatment escalation or intensified surveillance and a low residual-risk group in whom standard care may be sufficient, thereby reducing overtreatment and treatment-related toxicities. For NMIBC, it helps identify patients at higher risk who may warrant intensified or extended BCG maintenance and closer surveillance (Fig. 2C); for T1 NMIBC, it flags individuals likely to be BCG-unresponsive, supporting timely discussion of early cystectomy (Fig. 2E); and for MIBC, it highlights patients with elevated post-RC/chemotherapy recurrence risk who could be prioritized for adjuvant strategies aligned with emerging evidence for immunotherapy and antibody–drug conjugates (Fig. 2D) [30,31]. Because the score can be computed from routinely obtainable transcriptomic data, it is amenable to laboratory pipelines and potential electronic-health-record-linked dashboards for prospective auditing, multidisciplinary review, and shared decision-making. Overall, the integration of the 25-gene signature into current clinical practice addresses multiple unresolved clinical dilemmas, augmenting clinical decision-making across the entire BLCA treatment spectrum. From a research perspective, the signature provides a transparent and reproducible tool for prognostic enrichment, enabling stratified or randomized trial designs and balanced risk allocation across arms. This supports hypothesis generation for therapy response (e.g., drug sensitivity signals) and facilitates external benchmarking across cohorts, platforms, and analytical methods.

Medical therapy remains the cornerstone of advanced BLCA management. This study identified several FDA-approved anticancer agents as promising therapeutic candidates for different risk groups stratified by the signature. Notably, paclitaxel, which is already recommended by the European Association of Urology and National Comprehensive Cancer Network guidelines, emerged as an option for high-risk patient groups (Fig. 5C) [3,32]. The alignment between our results and established clinical recommendations underscores the clinical validity of this 25-gene signature. Moreover, other FDA-approved drugs, including lapatinib, gefitinib, ibrutinib, tamoxifen, and vorinostat, were computationally identified as candidate agents with predicted activity against high-risk tumors. Lapatinib, a dual epidermal growth factor receptor (*EGFR*)/human epidermal growth factor receptor 2 (*HER2*) inhibitor, exhibited significant efficacy in preclinical BLCA models and a modest effect in clinical trials [33,34]. Consistent with reports that AI-assisted HER2 scoring improves classification accuracy, the 25-gene signature could enhance the stratification of patients who are most likely to benefit from lapatinib [35]. Similarly, gefitinib, an EGFR-targeted therapy, has demonstrated promising preclinical outcomes and modest benefits in clinical trials (Fig. 5C) [36,37]. Irinotecan, alisertib, and niraparib were identified as potential options for low-risk patients (Fig. 5D). Notably, irinotecan combined with gemcitabine substantially enhances response rates and survival outcomes [38]. Alisertib and niraparib showed modest benefits in clinical trials, highlighting the necessity of precise patient selection [39,40]. Findings from the Genomics of Drug Sensitivity in Cancer (GDSC) should be treated as hypothesis-generating and validated through *in vitro*/*in vivo* studies, with future work incorporating cell sheet technology to enhance translational relevance [41]. In addition, our signature resolved TME heterogeneity: Low-risk tumors (Fig. 2B) and BCG-treated cohorts (Fig. 2E) showed heightened immune infiltration and improved immunotherapy responses, consistent with prior reports, including BCIPI, in which the immune-active (BCIPI-high) state exhibited greater infiltration and a higher likelihood of response [19,24,42]. In summary, our 25-gene signature offers exploratory signals that may inform risk-group-based treatment hypotheses, alongside multi-omics/AI integration for response prediction [28], advancing the field while acknowledging that real-world adoption will require transparent modeling and multicohort (including prospective) validation to address data heterogeneity, limited external validity, and interpretability [43].

Functional enrichment analysis of the 25-gene signature revealed the key molecular players: *FASN*, *SCD*, *SPINT1*, *MCM2*, and *CHMP4C*. Of these, *FASN* and *SCD*, which modulate lipid metabolism, are positioned as central nodes in oncogenic signaling networks. FASN-mediated palmitate synthesis fuels lipid biosynthesis, protein acylation, and oncogenic signaling pathways, such as the phosphatidylinositol 3-kinase (PI3K)/AKT/mechanistic target of rapamycin pathway in BLCA [44]. Similarly, SCD converts saturated fatty acids to monounsaturated fatty acids and regulates oncogenic signaling pathways, such as FGFR3 and PI3K/Akt/mTORC1 [45]. The other genes are involved in immune and inflammatory regulation (e.g., apolipoprotein B mRNA editing enzyme catalytic subunit 3H [*APOBEC3H*] and interferon regulatory factor 5 [*IRF5*]), cell–cell adhesion and extracellular matrix remodeling (cadherin 3 [*CDH3*], EGF containing fibulin extracellular matrix protein 1 [*EFEMP1*], and supervillin [*SVIL*]), nucleoside and small-molecule metabolism (solute carrier family 29 member 2 [*SLC29A2*], aldo-keto reductase family 1 member E2 [*AKR1E2*], and TCDD inducible poly(ADP-ribose) polymerase [*TIPARP*]), and developmental/morphological transcriptional control (transcription factor 15 [*TCF15*], zinc finger protein 135 [*ZNF135*], dysbindin domain containing 1 [*DBNDD1*], and chromosome 2 open reading frame 88 [*C2orf88*]). Comprehensive *in vivo* validation of all 25 genes was beyond the scope of this study; therefore, we prioritized *FASN* and *SCD* as exemplar lipid metabolic hubs for *in vitro* perturbation in BLCA cells. These assays support pathway-level, rather than direct causal, validation of the 25-gene model. Although *FASN* and *SCD* inhibition reduced colony formation and migration in BLCA cells, further CRISPR–Cas9 perturbation, rescue experiments, organoid models, and patient-derived xenografts are needed to clarify the causal hierarchy of this signature. Consistent with our findings, pharmacological inhibition or genetic silencing of *FASN* and *SCD* has been shown to significantly suppress BLCA proliferation and invasion in previous studies [46,47]. Although *FASN* and *SCD* are linked to tumorigenesis and prognosis in BLCA, the 25-gene signature provides a stronger prognostic value (Figs. 2 and 7A). In addition, drug sensitivity analysis of the 25-gene signature identified several drugs, including gefitinib, lapatinib, and paclitaxel (Fig. 5C), whose efficacies intersected with lipid metabolic pathways involving these enzymes in various cancers. Lapatinib effectively suppresses the HER2–PI3K/AKT–FASN signaling axis, thereby reducing tumor progression [48]. Gefitinib induces the down-regulation of SREBP1, a transcription factor that regulates *FASN* and *SCD* expression, further disrupting de novo lipogenesis and leading to reduced cell survival [49]. In addition, paclitaxel-based nanoparticles significantly reduce *FASN* expression and enhance chemotherapeutic efficacy, highlighting a potential therapeutic avenue [50]. These findings link drug response prediction with functional enrichment, highlighting the role of *FASN* and *SCD* in drug responses. Future studies should explore the combination of lipid metabolism or 25-gene-targeted inhibitors and current therapies and clarify the signature’s role in drug resistance to improve the treatment of BLCA.

This study analyzed a lipid-associated 25-gene signature in BLCA; however, several limitations must be noted. The study excluded nonurothelial and variant urothelial subtypes; accordingly, the findings are not directly applicable to these patients. Their divergent pathobiology and treatment paradigms warrant separate validation in variant-focused cohort studies. In addition, while the signature was validated across 4 independent retrospective datasets, its clinical utility remains unconfirmed in prospective patient cohorts, which is essential for real-world applications. We acknowledge that although feature selection was internally stabilized through repeated resampling within the random forest variable-hunting framework, a fully nested cross-validation procedure was not implemented in the discovery cohort. Consequently, the discovery set performance estimates may retain some optimism, and future studies should incorporate nested resampling or prospectively predefined training–validation designs to further minimize model selection bias. oncoPredict identified candidate drug response patterns that could guide future studies; however, these are computational, hypothesis-generating results that require *in vitro*/*in vivo* confirmation before any clinical application. While our functional validation confirmed the therapeutic potential of *FASN* and *SCD* inhibition, we acknowledge that the use of pharmacological inhibitors in cell lines may entail potential off-target effects compared to genetic perturbation methods. However, our findings align with those of previous studies demonstrating that genetic silencing (small interfering RNA/short hairpin RNA) of *FASN* and *SCD* suppresses BLCA progression [46,47]. Thus, our results serve as a translational proof of concept, reinforcing the clinical relevance of this signature. Future investigations should use CRISPR–Cas9-mediated editing and expand to diverse preclinical models (e.g., organoids or patient-derived xenografts) to fully characterize the mechanistic hierarchy of the 25-gene panel. Despite these limitations, this study presents substantial advancements. The 25-gene signature showed strong prognostic value, enhanced risk stratification, and may serve as a predictive tool for treatment response. When combined with clinical variables in a nomogram, it facilitates improved risk assessment and more personalized treatment strategies, thereby reducing the risk of over- and undertreatment.

## Materials and Methods

### Patient cohorts and data acquisition

Five publicly available BLCA datasets (TCGA BLCA, IMvigor210, GSE13507, GSE154261, and GSE48276), representing the full disease spectrum from nonmuscle-invasive to metastatic stages, were analyzed in this study. Analyses were confined to conventional urothelial carcinoma to ensure a uniform study population was obtained. Variant histologies, including micropapillary, squamous, glandular, nested, plasmacytoid, sarcomatoid, and small-cell/neuroendocrine, were excluded because of their divergent biological and clinical characteristics. The discovery set comprised the TCGA-BLCA cohort, which contained transcriptome data, methylome data, and deidentified clinical annotations for 408 tumors and 23 matched normal specimens. Primary level 3 transcriptomic (fragments per kilobase of transcript per million mapped reads, upper quartile normalized [FPKM-UQ], mapped to the hg19 reference genome) and methylomic data were retrieved from the National Cancer Institute Genomic Data Commons portal. DriverDB [51] was used for auxiliary multi-omics integration and annotation. The independent validation relied on 4 cohorts. Transcriptome data and curated clinical variables for the IMvigor210 study, which enrolled 298 patients with metastatic BLCA treated with atezolizumab, were obtained using the “IMvigor210CoreBiologies” R package [52]. The other 3 datasets were retrieved from the National Center for Biotechnology Information Gene Expression Omnibus database: GSE13507, GSE154261, and GSE48276. Cohort-level survival characteristics, including end-point definition, sample size, number of events, and median follow-up time within the prespecified 5-year analysis window, are summarized in Table S2.

### Construction of a prognostic gene signature through integrative transcriptomic and epigenomic analysis

Differential gene expression analysis was conducted on the TCGA-BLCA discovery set using DESeq2 (v1.40.2) [53]. Genes were considered differentially expressed if they met all 3 criteria: (a) Benjamini–Hochberg-adjusted *P* < 0.05; (b) |log_2_(fold change)| > 1; and (c) mean FPKM-UQ > 1000 across all tumor samples. This yielded 3,348 differentially expressed genes. Promoter DNA methylation *β* values (Illumina 450K array) were analyzed using MethylMix (v2.22.0) [54] to detect genes exhibiting at least one tumor-specific methylation state (adjusted *P* < 0.05). This procedure identified 2,296 differentially methylated genes.

Intersecting the differentially expressed and differentially methylated sets produced 494 genes with coordinated transcriptional and epigenetic dysregulation. These candidates were entered into a survival-oriented random forest model implemented in the randomForestSRC R package (v3.2.1) [55]. Forests were grown with 500 trees using the variable-hunting procedure implemented in the randomForestSRC::var.select() function. This procedure performs repeated internal 5-fold resampling, in which candidate variables are iteratively evaluated through forward selection within each training split, while the prediction error is assessed on the corresponding heldout fold. The variable selection frequency, average variable depth, and average model size were aggregated across all resampling iterations to identify stable candidate variables. The final signature was subsequently refitted to the full TCGA discovery cohort using the aggregated selected variables (Fig. S4).

To ensure the robustness of the signature across different quantification platforms (i.e., RNA sequencing and microarray), a systematic scoring method was applied to the data. First, the expression values for each of the 25 genes were *Z*-score-normalized within each cohort. Subsequently, a risk score was calculated for each patient using an unweighted directional sum approach, defined as

$$\text{Risk score}=\sum_{i=1}^{25}\left(\sigma_{i}\times Z_{i}\right)$$ (1)

where $Z_{i}$ represents the *Z*-score-normalized expression level of gene i and $\sigma_{i}$ represents the directionality coefficient (+1 for risk-associated genes and −1 for protective genes), determined on the basis of the univariate Cox regression hazard ratios in the discovery cohort.

We compared this unweighted approach with a coefficient-weighted model. Although the weighted model showed high performance in the discovery set, the unweighted directional sum demonstrated superior stability and reproducibility across independent validation cohorts. This parsimonious metric minimizes the risk of overfitting platform-specific technical noise, thereby facilitating broader clinical translation of the findings. The risk score distribution is presented in Fig. S5.

### Functional enrichment analysis

We conducted functional enrichment analysis using our in-house pipeline [56] to characterize the biological functions of the 25-gene signature. Overrepresentation analysis was performed against 2 pathway libraries: Kyoto Encyclopedia of Genes and Genomes [57] and Reactome [58]. For each pathway, a 2-tailed Fisher’s exact test quantified whether the proportion of 25 genes allocated to that pathway exceeded the proportion expected by chance, using the set of all genes that passed the initial expression filter (mean FPKM-UQ > 1) as a background. A *P* < 0.05 was considered significantly enriched.

### Nomogram development based on multivariate Cox regression analysis

A multivariate Cox proportional hazards regression model was used to develop a comprehensive prognostic tool for predicting survival outcomes. Initially, all potential predictors were evaluated using univariate analyses; those exhibiting statistical significance (*P* < 0.05) were subsequently incorporated into the multivariate analysis. For each selected variable, HRs and 95% confidence intervals were calculated to quantify the impact of these predictors on survival outcomes. Using the rms package [50], a nomogram was constructed by translating the regression coefficients into a point scale, enabling each predictor to contribute proportionally to the total risk score. This total score is then correlated with the predicted probabilities of survival at specified time intervals, such as 1-, 3-, and 5-year survival rates. Patients with incomplete clinical annotations required for multivariable modeling were excluded using complete case analysis, and no imputation was performed.

### Nomogram performance evaluation

A nomogram was constructed based on a parametric Weibull survival model using the rms package in R. The model included the lipid-related signature and clinically relevant variables (age at diagnosis, gender, pathologic stage, anatomic neoplasm subdivision, diagnosis subtype, and histologic grade). The discriminative ability of the nomogram was assessed using Harrell’s concordance index (C-index), with bootstrap validation (*B* = 200) to obtain optimism-corrected estimates of the area under the curve. Calibration was evaluated using calibration plots at 5-year intervals. Calibration curves were generated using the calibrate() function, using the Kaplan–Meier method for observed survival probabilities and 200 bootstrap resamples to calculate optimism-corrected estimates. To evaluate the clinical utility of the prognostic model, decision curve analysis was performed using the dcurves package in R. The model was used to calculate the predicted 5-year event (mortality/recurrence) probability for each patient, derived from the estimated cumulative baseline hazard at 60 months. The net benefit was calculated across a continuous range of threshold probabilities (1% to 100%) and compared with 2 default clinical management strategies: treat all (intervening for all patients regardless of risk score) and treat none (intervening for no patients). A 2-sided *P* < 0.05 was considered statistically significant.

### *In silico* prediction of drug sensitivity

Individualized drug response profiles were derived from tumor transcriptomes using the oncoPredict R package (v1.4.1) [59], which uses ridge regression models trained on the GDSC2 cell line panel. Gene identifiers in each BLCA cohort were initially converted to HUGO Gene Nomenclature Committee (HGNC) symbols and restricted to the intersection of the 16,382 genes present in the GDSC2 training matrix. Expression values were log_2_-transformed, mean-centered, and variance-scaled in accordance with the internal prepareInput() function to ensure compatibility with pretrained models. For each patient, oncoPredict generated a predicted IC_50_ for 198 anticancer drugs. Lower IC_50_ values indicate greater predicted sensitivity, whereas higher values indicate resistance. Group-wise differences in drug response predictions between high- and low-risk patients (defined by the 25-gene score) were evaluated using 2-sided Wilcoxon rank sum tests. Compounds with a nominal *P* < 0.05 were considered differentially predicted between risk groups and prioritized as computational, hypothesis-generating signals for subsequent biological interpretation. These *in silico* predictions require experimental confirmation and are not intended as direct clinical sensitivity claims.

### Estimation of tumor-infiltrating immune cells

The composition of tumor-infiltrating immune cells was deduced from bulk RNA sequencing profiles using several bioinformatics tools, including CIBERSORT [60], XCELL [61], TIMER [62], ImmuCellAI [63], and ESTIMATE [64]. CIBERSORT uses a deconvolution algorithm based on support vector regression and predefined gene signatures to estimate the composition of tumor immune cells, thereby providing a comprehensive view of the immune microenvironment of the tumor. XCELL infers immune cell types through gene signature enrichment analysis, which is trained on pure cell transcriptomes and reduces interference between similar cell types to enhance accuracy. TIMER quantifies immune cell abundance using a statistical deconvolution method and selects genes associated with lower tumor purity for modeling. ImmuCellAI identifies immune cell types through a hierarchical gene set signature approach and enrichment analysis that simulates flow cytometry, offering precise insights into the tumor–immune landscape. ESTIMATE utilizes single-sample gene set enrichment analysis to calculate stromal and immune scores based on specific gene expression signatures; these scores are aggregated to derive the ESTIMATE score, which is then used to infer tumor purity. To investigate the relationship between the immune landscape and our 25-gene risk model, multivariate logistic regression was conducted to analyze patient risk stratification and each infiltration score, with tumor purity as the confounding variable. Associations with a nominal *P* value of less than 0.05 were considered significant.

### Cell lines

Human BLCA cell lines 5637 (COSMIC ID COSS687452) and J82 (COSMIC ID COSS753566) were obtained from the Taiwan BioMedicine Culture Collection. According to the Cell Model Passports, both cell lines exhibit microsatellite stability; the 5637 line possesses a *TP53* p.R280T mutation alongside a wild-type *FGFR3*, whereas the J82 line contains *TP53* p.E271K/p.K320N mutations and activating *FGFR3* p.K651E/p.L309I variants. The cells were cultured in minimum essential medium supplemented with 10% fetal bovine serum and 1% penicillin/streptomycin and maintained at 37 °C in a humidified incubator with 5% CO₂, in accordance with American Type Culture Collection guidelines.

### Cell viability/proliferation assay

To assess the viability of J82 and 5637 cells transfected with synthetic mimics or inhibitors, WST-8, a water-soluble tetrazolium salt reduced by cellular dehydrogenase activity, was administered on days 3 and 5. The resultant formazan dye, which correlates directly with the number of viable cells, was quantified by measuring the optical density at 450 nm.

### Doubling time assay

To ascertain the doubling time intervals of various cell types, a straightforward protocol was used in an adherent culture. Initially, each cell type was seeded at a density of 20,000 cells/ml in supplemented Dulbecco’s modified Eagle’s medium in a plate (day 0). The plates were subsequently incubated under standard laboratory conditions for approximately 1 week. During the incubation period, the cells were exposed to different concentrations of lipids (0, 2, 4, 6, 8, 10, and 12 mM) for specified time intervals (24, 48, 72, and 96 h). To ensure the uninterrupted growth of the adherent (active) cell population, the cell culture medium was carefully replaced as needed. Every 24 h postseeding, the culture medium from one well per cell type was aspirated, and a 1× trypsin–EDTA solution (Sigma-Aldrich, Germany) was applied for 1 to 2 min. Subsequently, the mixture was gently triturated for 1 min to obtain a single-cell suspension. The entire content of each well was transferred to a 2-ml Eppendorf tube. To permanently fix the cells, 4% formaldehyde was added to the tube, which was then stored in a refrigerator for future analysis. This procedure was repeated until 100% cell confluence was attained. The cell concentration of each cell type was measured at 24-h intervals using a hemocytometer.

### Colony formation assay

In summary, 5,637 cells (1,000 cells per well) and J82 cells (500 cells per well) were seeded into 6-well plates and incubated at 37 °C for 24 h. The following day, one group served as the control and was maintained in the growth medium, while the experimental group received an addition of 50 μM FASN inhibitor (MedChemExpress, TVB-3664, catalog no. HY-120062) and SCD inhibitor (MedChemExpress, A939572, catalog no. HY-50709) to the growth medium and was cultured for a duration of 13 d. Subsequently, the cells were fixed with 4% formaldehyde (Sigma-Aldrich) and stained with 0.5% crystal violet (Sigma-Aldrich). Finally, colonies were enumerated directly on the culture dishes, images of the wells were captured using an imager (ChemiDoc XRS+ System with Image Lab software #1708265), and colony numbers were quantified using ImageJ.

### Migration and invasion assay

Cells were cultured in Millicell tissue culture plate well inserts with 8-μm pores for 12 h to evaluate their migratory capacity. For invasion analysis, cells were seeded onto BD BioCoat Matrigel Invasion Chambers and allowed to invade for 20 h. Subsequently, the migrated or invaded cells were fixed with methanol for 10 min and stained with crystal violet for 1 h. The total number of stained cells was observed and recorded using a light microscope.

### Statistical analysis

Group differences were evaluated using parametric or nonparametric methods, selected on the basis of data distribution and measurement scale. The IC_50_ values were compared between the 25-gene high- and low-risk groups utilizing the Wilcoxon rank sum test (wilcox.test). Variation in *FASN* and *SCD* expression across American Joint Committee on Cancer (AJCC) stages I to IV (Fig. 6B) was assessed using one-way ANOVA. When the omnibus *F*-test indicated significance, Tukey’s honestly significant difference test was used for pairwise comparisons. *In vitro* colony formation and migration data (Fig. 6C and E) were analyzed using 2-tailed unpaired Student’s *t* tests. Statistical significance was set at *P* < 0.05.

## Ethical Approval

Not applicable.

## Data Availability

All data pertinent to this article are presented within the manuscript and are exhibited in various figures and tables. The signature identification code used in this study is available at GitHub (https://github.com/BioinfOMICS/BLCA). Further details are available from the corresponding author upon reasonable request.
