# Supplementary material for: Epigenomics-Guided Multi-Omics Integration Uncovers a Lipid-Metabolic Signature with Translational Utility in Bladder Cancer
Source: Comput Struct Biotechnol J. 2026 Jul 24;35(1):0139. doi: 10.34133/csbj.0139 (PMC13396492; doi:10.34133/csbj.0139)
Supplement: Supplementary 1 — Figs. S1 to S5 Tables S1 and S2 [file csbj.0139.f1.zip › Supplementary Figure_20260518.pdf]

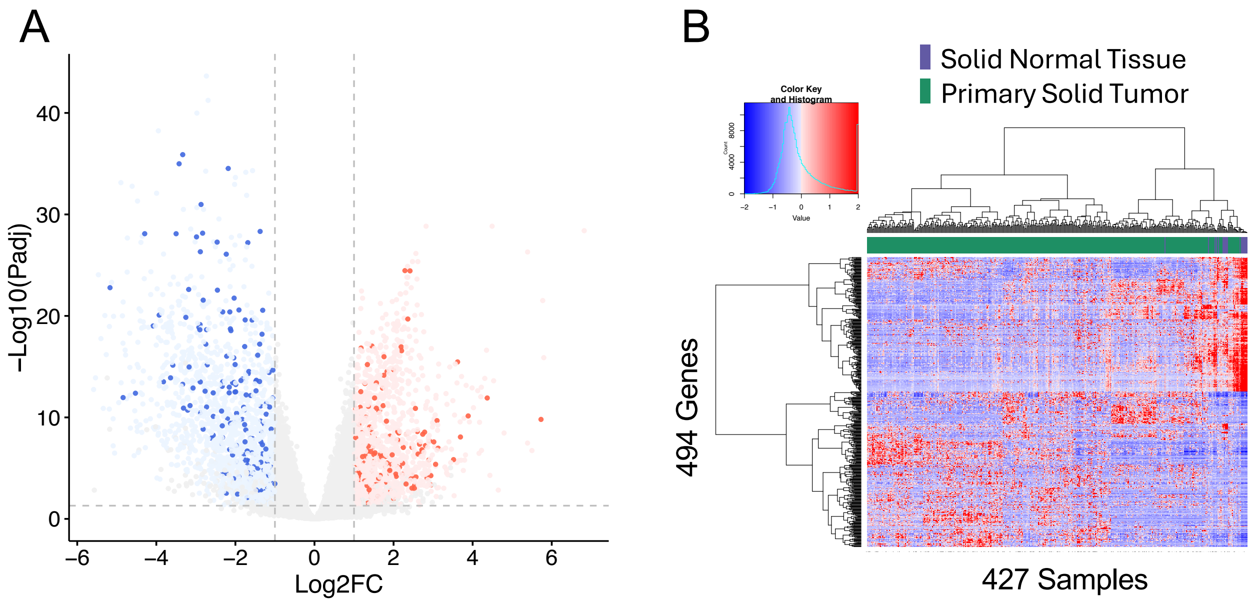

Supplementary Figure 1. Differential gene expression between tumor and normal bladder tissues in the TCGA-BLCA cohort.

(A) Volcano plot illustrating significantly upregulated and downregulated genes (Padj < 0.05) between primary solid tumors and solid normal tissues. **(B)** Hierarchical clustering heatmap of the 494 differentially expressed genes, demonstrating clear separation between tumor and normal samples across 427 TCGA-BLCA specimens. Color bars indicate sample type (green: primary tumor; purple: solid normal tissue).

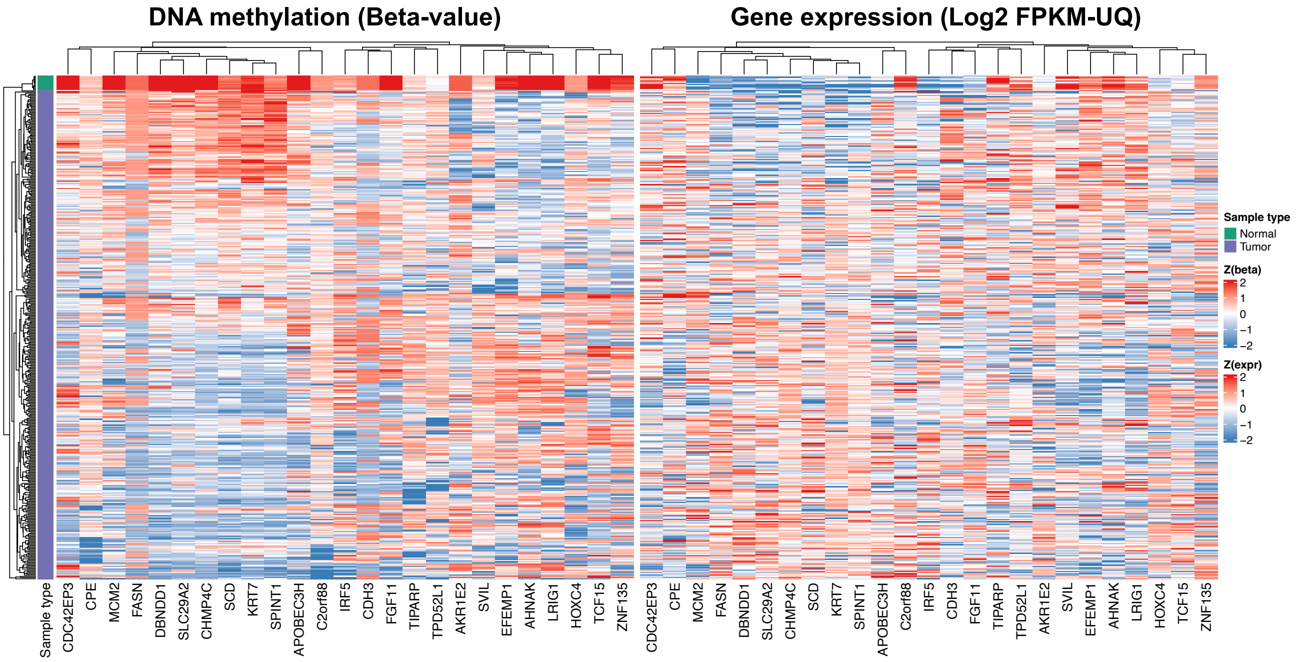

Supplementary Figure 2. Matched heatmaps of promoter DNA methylation and gene expression profiles for the 25-gene signature.

Side-by-side heatmaps illustrate promoter methylation (β-values) and log2-transformed gene expression across matched TCGA-BLCA samples, using identical gene and sample ordering. The majority of signature genes display clear inverse methylation–expression patterns, consistent with promoter-associated transcriptional repression.

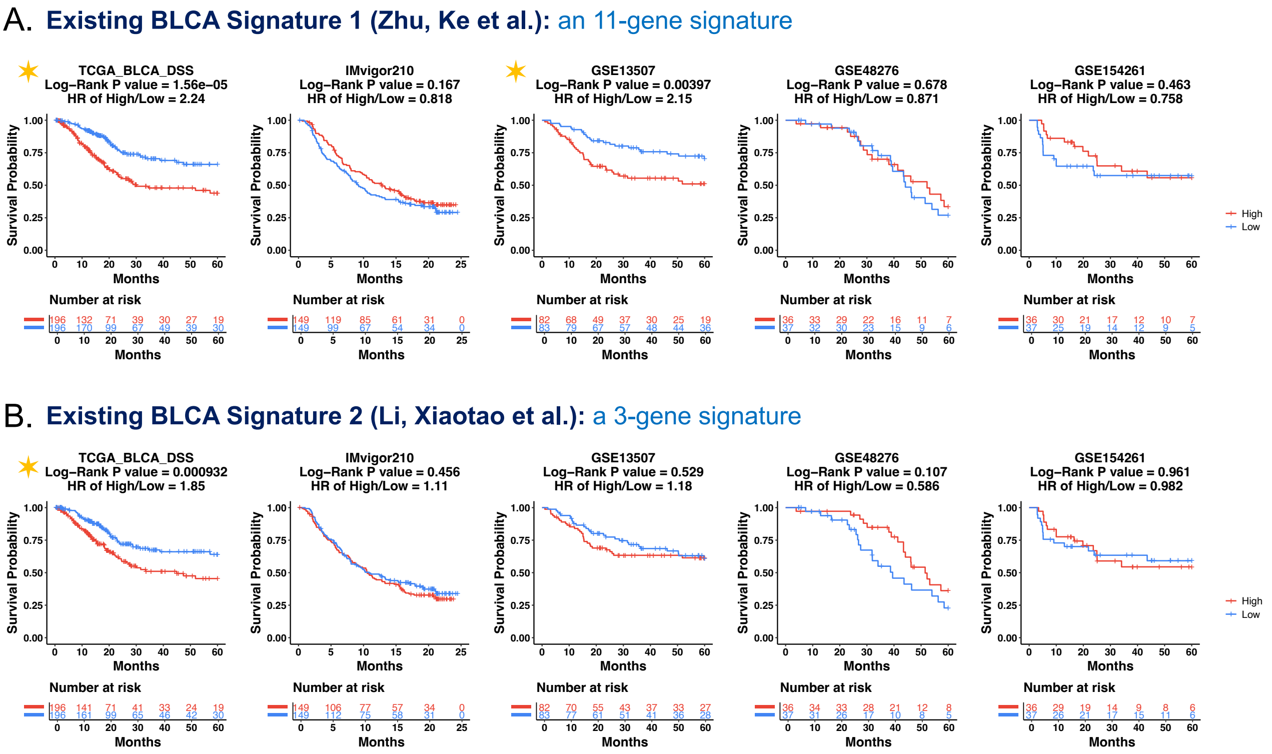

Supplementary Figure 3. Comparison of prognostic performance between the proposed 25-gene signature and previously published BLCA signatures.

Kaplan–Meier survival curves showing the performance of two existing bladder cancer prognostic signatures **(A)** the lipid metabolism–related signature by Zhu et al. and **(B)** the metabolism-related signature by Li et al. across five independent cohorts (TCGA-BLCA, IMvigor210, GSE13507, GSE48276, and GSE154261). In contrast to the inconsistent or cohort-restricted performance of published signatures, our 25-gene signature demonstrated superior discrimination and robust validation across all datasets. Yellow stars indicate datasets where the corresponding signature achieved statistically significant survival separation (log-rank $p < 0.05$).

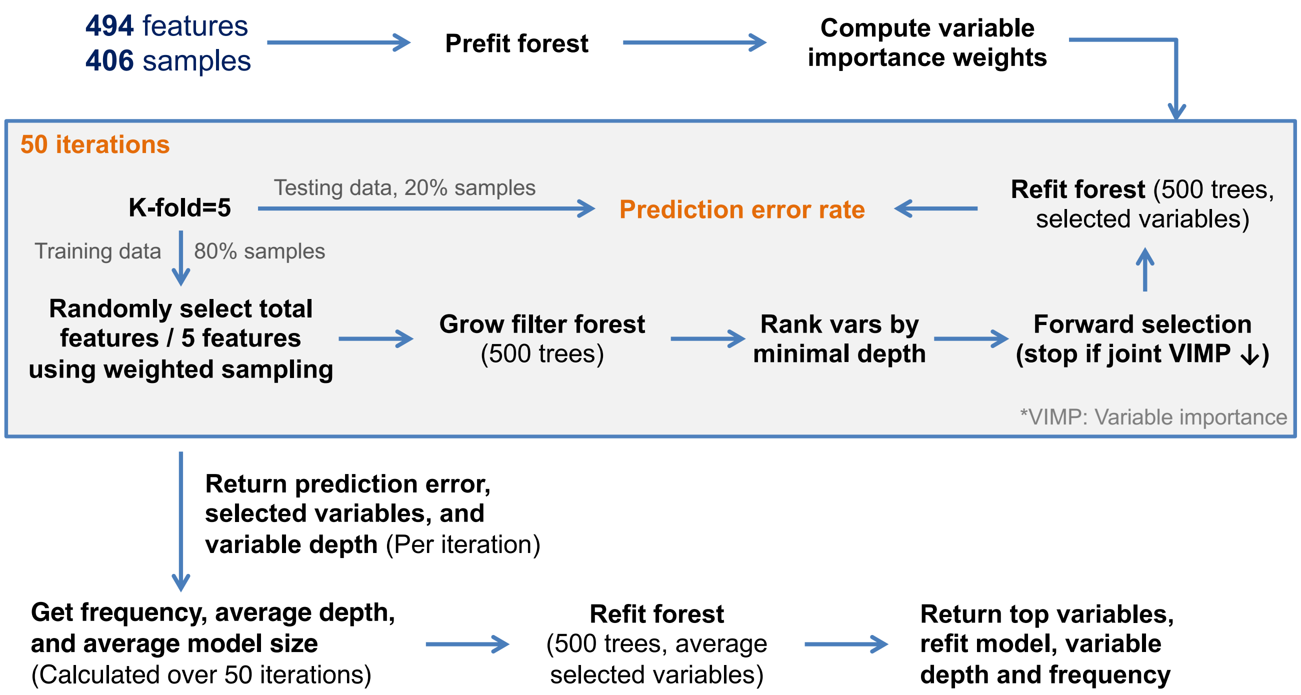

Supplementary Figure 4. Overview of the random forest–based feature selection workflow.

The procedure integrates repeated subsampling, K-fold cross-validation, weighted feature sampling, minimal-depth–based variable ranking, and forward selection with a variable-importance stopping rule. Across 50 iterations, each cycle uses 80% of samples for training and 20% for testing to estimate prediction error and prevent overfitting. Features selected consistently across iterations are used to refit the final random forest model.

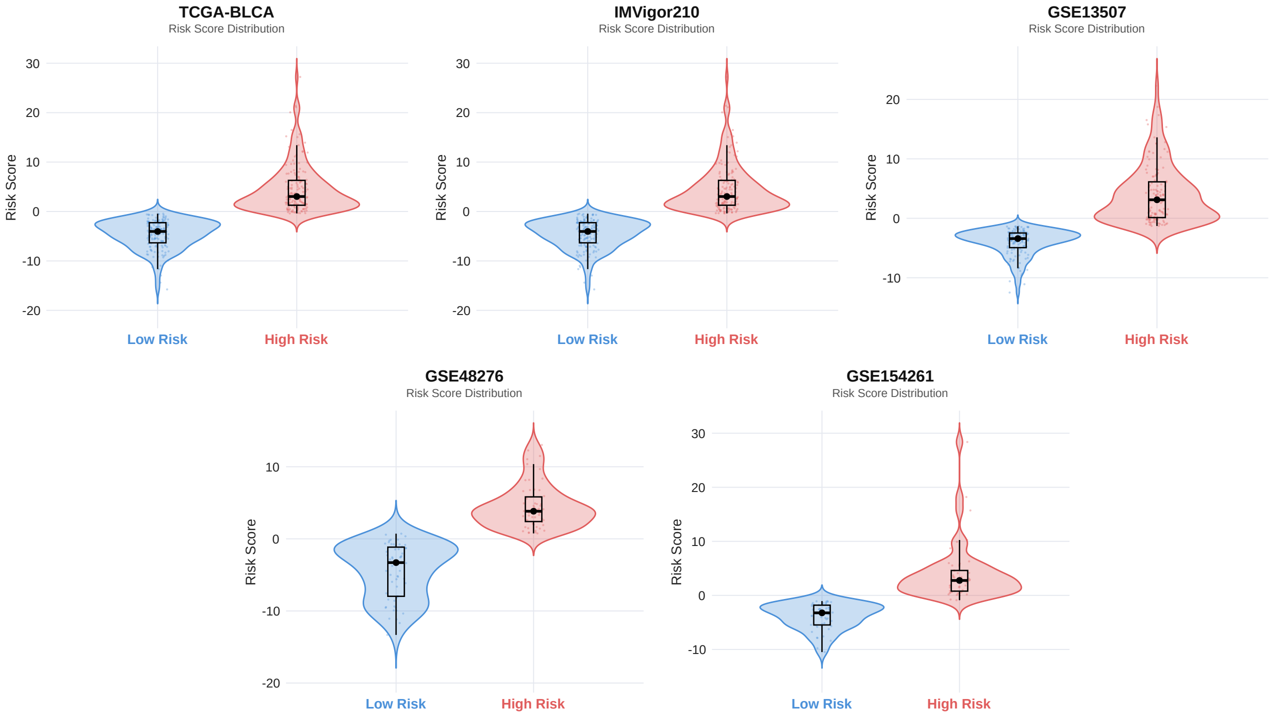

Supplementary Figure 5. Risk score distributions across the training and validation cohorts.

Violin and box plots showing the distribution of the 25-gene signature–derived risk scores between high-risk and low-risk groups in five independent cohorts: TCGA-BLCA, IMvigor210, GSE13507, GSE48276, and GSE154261. In all datasets, high-risk samples exhibit significantly higher risk scores compared to low-risk samples, demonstrating clear group separation and supporting the robustness of the stratification used in the survival analyses.
